# Disruption of Autographa Californica Multiple Nucleopolyhedrovirus *ac111* Results in Reduced *per os* Infectivity in a Host-Dependent Manner

**DOI:** 10.3390/v10100527

**Published:** 2018-09-27

**Authors:** Sainan Li, Lu Li, Haizhou Zhao, Wenhua Liu

**Affiliations:** 1 Department of Biology, Zhaoqing University, Zhaoqing 526061, China; zhaohaizhou012@163.com (H.Z.); wenhualiu@hotmail.com (W.L.); 2 State Key Laboratory of Biocontrol, Sun Yat-sen University, Guangzhou 510275, China; ll627328115@163.com

**Keywords:** AcMNPV, *ac111*, BV, ODV, *per os* infectivity

## Abstract

The Autographa californica multiple nucleopolyhedrovirus (AcMNPV) *ac111* gene is highly conserved in lepidopteran-specific baculoviruses, and its function in the AcMNPV life cycle is still unknown. To investigate the function of *ac111*, an *ac111*-knockout AcMNPV (vAc111KO) was constructed through homologous recombination in *Escherichia coli*. Viral growth curve analysis and plaque assays showed that the deletion of *ac111* had no effect on infectious budded virion production. Quantitative real-time polymerase chain reaction analysis confirmed that viral DNA replication was unaffected in the absence of *ac111*. Electron microscopy revealed that the *ac111* deletion did not affect nucleocapsid assembly, occlusion-derived virion formation, or the embedding of occlusion-derived virions into the occlusion bodies. However, in vivo bioassays showed that although the deletion of *ac111* did not affect the *per os* infectivity of AcMNPV in *Spodoptera exigua* larvae, it led to an approximately five-fold reduction in infectivity of AcMNPV in *Trichoplusia ni* larvae, and vAc111KO took approximately 21 h longer to kill *Trichoplusia ni* larvae than the wild-type viruses. Taken together, our results demonstrated that although *ac111* is not essential for virus replication in vitro, it plays an important role in the *per os* infectivity of AcMNPV in a host-dependent manner.

## 1. Introduction

Baculoviruses are a family of rod-shaped, enveloped, double-stranded DNA viruses which are specifically pathogenic to invertebrate species, mainly belonging to the orders Lepidoptera, Hymenoptera, and Diptera [1]. *Baculoviridae* consists of *Alphabaculovirus*, *Betabaculovirus*, *Gammabaculovirus,* and *Deltabaculovirus* [2]. Based on phylogenetic analysis of the *polyhedrin* (*polh*) gene, *Alphabaculovirus* can be further subdivided into Group I and Group II nucleopolyhedroviruses (NPVs) [3]. Autographa californica multiple NPV (AcMNPV) is the archetype of the *Baculoviridae* family and the first baculovirus to be completely genome-sequenced [4]. The AcMNPV genome is approximately 134 kbp and encodes 154 potential open reading frames (ORFs) [4]. To date, more than 80 baculovirus genomes have been completely sequenced, and there are 38 genes conserved in all these genomes. These 38 common genes are considered to be the baculovirus core genes [1,5,6,7].

During the baculovirus infection cycle, two morphologically distinct but genetically identical progeny virion phenotypes, budded virions (BVs), and occlusion-derived virions (ODVs), are produced. Although BVs and ODVs are similar in their nucleocapsid structure, they differ in their origin and the composition of their envelopes, which reflects their distinct roles in the baculovirus life cycle [8,9]. In the early infection phase, newly assembled nucleocapsids egress from the nucleus, migrate across the cytoplasm, and bud from the plasma membrane to form BVs. BVs are responsible for spreading infections among susceptible insect tissues [10]. During the late phase of infection, ODVs are formed and are subsequently embedded in the paracrystalline protein matrix to form occlusion bodies (OBs). ODVs can initiate primary infection in the insect hosts [9,11].

The site of baculovirus primary infection is the midgut of host insects. Upon oral ingestion, the OBs are dissolved under alkaline conditions in the insect midgut, and they release ODVs, which then penetrate the peritrophic matrix and infect epithelial cells. A number of ODV envelope-associated proteins that play important roles in oral infectivity are designated as *per os* infectivity factors (PIFs), including P74, and PIF-1 to 8 [6,12,13,14,15,16,17,18,19,20,21,22,23]. In addition, the Ac108 homolog in *Spodoptera frugiperda* NPV, Sf58, which is only conserved in lepidopteran baculoviruses, has been shown to be a PIF of lepidopteran-infecting baculoviruses [24]. Moreover, some other less-conserved proteins, such as Ac18, Ac32, ODV-E66, Ac114, Ac124, Ac145, and Ac150 have been shown to be involved in the oral infection of AcMNPV [25,26,27,28,29,30,31]. *ac18* is a highly conserved gene in lepidopteran NPVs, though *ac18* deletion did not affect viral propagation in vitro or AcMNPV infectivity for *Trichoplusia ni* larvae; however, the LT_50_ was 24 h longer for the *ac18* knockout virus in *T. ni* larvae compared to the wild-type virus [28]. Previous studies have also demonstrated that although *ac114* and *ac124* are nonessential for AcMNPV replication in vitro, *ac114* and *ac124* selectively affect viral infectivity and viral killing speed, respectively, of AcMNPV in *S. exigua* larvae [27,29]. Therefore, baculovirus *per os* infection is a complex process involving numerous virus proteins coordinating the establishment of the primary infection.

The AcMNPV *orf111* (*ac111*) is located between AcMNPV nucleotides (nt) 101,624 and 101,827 [4], and its homologs are present in all sequenced Group I NPVs, certain Group II NPVs, and granuloviruses (GVs). Transcriptional analysis identified that *ac111* is transcribed as an early gene [32]. Recently, the Ac111 homolog in *Bombyx mori* NPV, Bm93, has been found not to be essential for virus replication [33]. However, currently the role of *ac111* in the AcMNPV life cycle is still unknown.

In this study, an *ac111* knockout AcMNPV (vAc111KO) was constructed to investigate the role of *ac111* in the AcMNPV life cycle. We found that the *ac111* deletion had no significant effect on infectious BV production, viral DNA replication, or viral morphogenesis. However, the oral infectivity bioassays showed that, in *T. ni* larvae, the infectivity of vAc111KO was reduced by about five times relative to the wild-type virus, and the mutant virus took approximately 21 h longer to kill these larvae. Interestingly, the deletion of *ac111* did not affect the infectivity of AcMNPV in another lepidopteran host, *S. exigua* larvae. Therefore, our results suggest that Ac111 plays an important role in virus *per os* infectivity in a host-dependent manner.

## 2. Materials and Methods

### 2.1. Computer-Assisted Analysis

HHpred analysis [34], NCBI’s Conserved Domain Database [35], Phrye [36], and SMART [37] were used to predict the conserved domains of Ac111. The Position-Specific Iterated BLAST algorithm was used to search the homologues of Ac111 in the NCBI database. ClustalX [38] and GeneDoc [39] were used to perform and edit multiple sequence alignments, respectively.

### 2.2. Cell Lines, Viruses, and Insect Larvae

The Sf9 cell line and Se301 cell line [40], which are derived from the fall armyworm *Spodoptera frugiperd* and *S. exigua*, respectively, were cultured at 27 °C in TNM-FH medium (Invitrogen, Carlsbad, CA, USA) supplemented with 10% fetal bovine serum, penicillin (100 μg/mL), and streptomycin (30 μg/mL). The bMON14272 bacmid (Invitrogen, Carlsbad, CA, USA), which contains the AcMNPV genome, was propagated in DH10B cells as previously described [41]. *T. ni* and *S. exigua* larvae were reared on an artificial diet at 28 °C [42]. BV titers were determined using a 50% tissue culture infective dose (TCID_50_) endpoint dilution assay in Sf9 cells or Se301 cells, as previously described [43].

### 2.3. Construction of an ac111-Knockout Virus

Employing the bacmid bMON14272, an *ac111*-knockout AcMNPV bacmid (bAc111KO) was generated through ET homologous recombination in *Escherichia coli*, as previously described [44]. Briefly, a 400-bp fragment that was homologous to the 5′ region of the *ac111* ORF (AcMNPV nt 101,822 to 102,221) was PCR-amplified from bMON14272 using the primers ac111PF1/ac111PR1 (the PCR primers used in this study are listed in Table 1). A 340-bp fragment homologous to the 3′ region of the *ac111* ORF (AcMNPV nt 101,402 to 101,741) was PCR-amplified from bMON14272 using the primers ac111PF2/ac111PR2. The 5′- and 3′-PCR products were digested with *Pst*I/*Hind*III and *Sac*I/*Bam*HI, respectively, and ligated into pUC18-Cm [45], which contains a chloramphenicol resistant (*Cm*) gene cassette to generate pUC18-111US-Cm-111DS. The plasmid was then digested with SacI and *Hind*III to obtain a linear 1778-bp fragment in which the *Cm* gene cassette was flanked by *ac111* 5′- and 3′-regions. Homologous recombination between the deletion region of *ac111* (AcMNPV nt 101,742 to 101,821) and the *Cm* cassette in the linear fragment was performed to obtain an *ac111*-knockout bacmid, bAc111KO. The replacement in bAc111KO was confirmed by PCR and sequencing.

To facilitate the detection of viral infection, the pFB1-PH-GFP donor plasmid, containing the AcMNPV *polh* gene and the *enhanced green fluorescence protein* (*egfp*; referred to as *gfp* in the present study) gene, was transformed into electrocompetent DH10B cells harboring the bAc111KO bacmid and the pMON7124 helper plasmid as previously described [45], to generate the *ac111*-knockout virus, vAc111KO. To generate the *ac111-*repaired virus, vAc111:HA, the donor plasmid pFB1-Ac111:HA-PH-GFP was constructed using the following steps. The 531-bp DNA fragment containing the promoter and the ORF of *ac111* tagged with an HA epitope prior to the stop codon was PCR amplified from bMON14272 using the primers ac111PF3/ac111PR3, and it was cloned into the SacI/BamHI-digested pUC18-SV40 plasmid [46] to construct the pUC18-Ac111:HA plasmid. The Ac111:HA fragment was digested from pUC18-Ac111:HA with *Sac*I/*Xba*I, and subcloned into *Sac*I/*Xba*I-digested pFB1-PH-GFP to generate the donor plasmid pFB1-Ac111:HA-PH-GFP. The pFB1-Ac111:HA-PH-GFP was then transformed into electrocompetent DH10B cells harboring the bAc111KO bacmid and the helper plasmid pMON7124 to generate the *ac111-*repaired virus, vAc111:HA. Similarly, vAcWT, a wild type virus, was also constructed by inserting *polh* and *gfp* genes into bMON14272.

### 2.4. Viral Propagation Analysis

Sf9 cells (1.0 × 10^6^) were transfected with 1.0 μg bacmid DNA using the Cellfectin liposome reagent (Invitrogen Life Technologies, Carlsbad, CA, USA). For infection, Sf9 cells (1.0 × 10^6^) or Se301 cells (1.0 × 10^6^) were infected with BVs at a multiplicity of infection (MOI) of 5 TCID_50_/cell. At the designated time points post transfection (p.t.) or post infection (p.i.), the supernatants containing BVs were harvested and titered. To further evaluate the effect of the *ac111* deletion on BV production, a plaque assay was performed as described previously [43,47].

### 2.5. Real-Time PCR (qPCR) Analysis

qPCR analysis was performed to assess the effect of the *ac111* deletion on viral DNA synthesis as described previously [7,46].

### 2.6. Transmission Electron Microscopy (TEM)

1.0 × 10^6^ Sf9 cells were transfected with 1.0 μg bacmid DNA of vAc111KO, vAc111:HA, or vAcWT. At 72 h p.t., the cells were dislodged with a cell scraper, pelleted at 1000 × g for 10 min, and prepared for TEM, as previously described [7]. The samples were observed with a JEOL JEM-1400 transmission electron microscope (Tokyo, Japan) at an accelerating voltage of 120 kV.

### 2.7. Bioassays in T. ni and S. exigua Larvae

In order to determine whether the *ac111* deletion had any effect on the infectivity of AcMNPV in insect larvae, bioassays were performed using *T. ni* or *S. exigua* larvae, as described previously [28]. Briefly, OBs and BVs were obtained from vAc111KO-, vAc111:HA-, or vAcWT-infected Sf9 cells, as previously described [48]. Different doses of BVs were hemocoelically injected into fourth-instar *T. ni* or *S. exigua* larvae to examine the infectivity of BVs in vivo. H_2_O was used as a blank control. For the oral infectivity bioassays, the OBs of vAc111KO, vAc111:HA, or vAcWT purified from infected Sf9 cells, were resuspended in double-distilled water and administered to newly molted *T. ni* or *S. exigua* larvae via oral inoculation. To determine the median lethal dose (LD_50_), 1 μL water (control) or 1 μL OB suspensions of each virus was administered to a small piece of diet of newly molted third-instar *T. ni* or *S. exigua* larvae, respectively. For each virus in *T. ni*, the doses were 9, 30, 90, 3 × 10^2^, and 9 × 10^2^ OBs per larva. For each virus in *S. exigua,* the doses were 9 × 10^2^, 3 × 10^3^, 9 × 10^3^, 3 × 10^4^, and 9 × 10^4^ OBs per larva. Larvae were reared individually in 24-well plates until all of the diet was consumed, then fresh diet was added. Only the larvae that completely ingested the diet with OBs within 12 h were further reared and monitored daily, until all larvae had either pupated or died. The median lethal time (LT_50_) was determined using the droplet feeding method [49]. The newly molted third-instar *T. ni* or fourth-instar *S. exigua* larvae were starved for 6 h, and then they were fed with 1 μL OB suspensions of each virus with concentrations of 1 × 10^7^ or 1 × 10^8^ OBs/mL, respectively. For each virus, the final mortalities were about 99% or 86% in *T. ni* or *S. exigua*, respectively. The larvae that have ingested virus were reared individually and the mortality was recorded every 12 h until all larvae had either pupated or died. The experiments with each dose in both oral and injection treatments were performed three times (30 larvae per dose). The LD_50_ value and the LT_50_ value were determined by probit analysis and the Kaplan-Meier estimator, respectively.

## 3. Results

### 3.1. Sequence Analysis of Ac111 and Its Homologues

**Figure 1:**
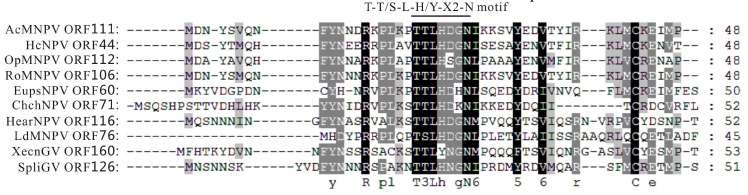
Multiple-sequence alignment of 10 selected Ac111 homologues. The alignment was performed and edited using Clustal X and GeneDoc, respectively. The Ac111 homologue sequences used are as follows: NP_054141.1 for AcMNPV ORF111, YP_473232.1 for HcNPV ORF44, NP_046268.1 for OpMNPV ORF112, AAN28146.1 for RoMNPV ORF106, YP_002854670.1 for EupsNPV ORF60, YP_249675.1 for ChchNPV ORF71, NP_203672.1 for HearNPV ORF116, NP_047713.1 for LdMNPV ORF76, NP_059308.1 for XecnGV ORF160, and YP_001257077.1 for SpliGV ORF126. Black, dark gray, or light gray shading denote 100%, 80%, or 60% conservation, respectively. The predicted T-T/S-L-H/Y-X_2_-N motif found in all of the homologues is indicated.

Homologues of *ac111* are present in lepidopteran-specific baculoviruses. Sequence-based searches have shown that Ac111 belongs to the Baculo-8kDa superfamily with no homology to other identified functional domains. The alignment of Ac111 orthologs from several selected lepidopteran-specific baculovirus groups, including AcMNPV, Hyphantria cunea NPV (HcNPV), Orgyia pseudotsugata MNPV (OpMNPV), Rachiplusia ou MNPV (RoMNPV), Euproctis pseudoconspersa NPV (EupsNPV), Chrysodeixis chalcites NPV (ChchNPV), Helicoverpa armigera NPV (HearNPV), Lymantria dispar MNPV (LdMNPV), Xestia c-nigrum GV (XecnGV), and Spodoptera litura GV (SpliGV), revealed that a T-T/S-L-H/Y-X_2_-N motif (where X was any other amino acid) was conserved in all Ac111 homologues (Figure 1). Currently, no information about this motif in the function of *ac111* is available and further studies need to be undertaken to determine its importance.

### 3.2. Construction of an ac111-Knockout AcMNPV

**Figure 2:**
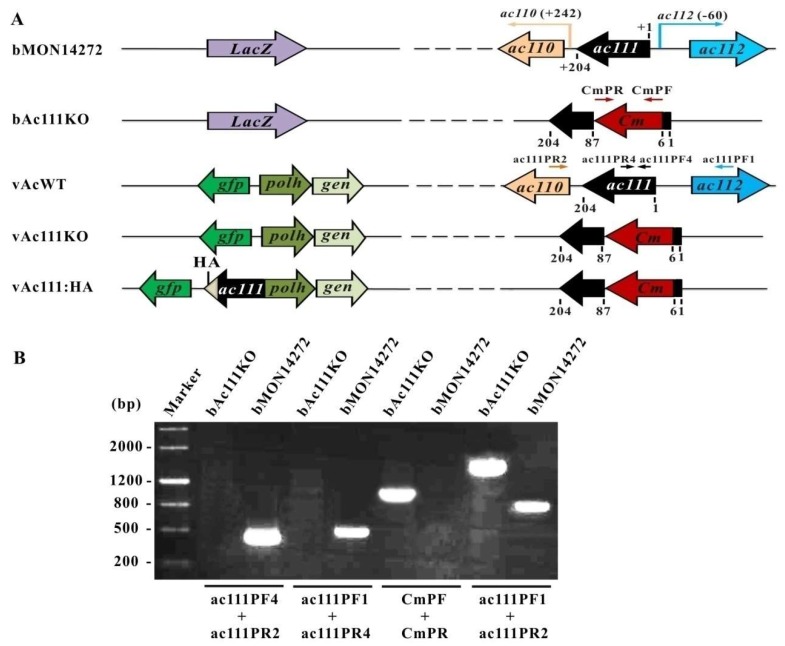
Construction of recombinant viruses and their confirmation. (**A**) Construction of the recombinant viruses. The *ac111* ORF has 204 nt. The first nucleotide of the initiation codon ATG of *ac111* is indicated as +1. The 5′ transcription start site of *ac110* is located 242 nt downstream of the 5′ end of *ac111*, and the 5′ transcription start site of *ac112* is located 59 nt upstream of the initiation codon ATG of *ac111*. The *ac111* deletion bacmid, bAc111KO, was generated by replacing an 80-bp fragment between the 6 and 87 nt of *ac111* in the bMON14272 genome with a 1038-bp *Cm* cassette via ET homologous recombination. The *polh* and *gfp* genes were inserted into the *polh* locus of bAc111KO by Tn7-mediated transposition to construct the *ac111* deletion virus, vAc111KO. The rescue virus, vAc111:HA, was constructed by inserting the *ac111* gene under the control of its native promoter with an HA tag (indicated as a grey triangle) prior to the *ac111* stop codon, together with the *polh* and *gfp* genes, into the *polh* locus of bAc111KO. The *polh* and *gfp* genes were inserted into bMON14272 to generate the wild-type control virus, vAcWT. The approximate locations of primers (arrows) used in the confirmation of the disruption of *ac111* and the correct insertion of the *Cm* gene cassette are shown in the diagram; (**B**) Confirmation of bacmids by PCR. Bacmid DNA templates are shown above each lane, and the primer pairs used are shown below. The migration of DNA markers is shown to the left in basepairs (bp).

Based on AcMNPV transcriptomics [32], the 5′ transcription start site of *ac110* is located 38 nt downstream of the 3′ end of *ac111*, and the 5′ transcription start site of *ac112* is located 59 nt upstream of the initiation codon ATG of *ac111*. To investigate the role of Ac111 in the baculovirus life cycle, the *ac111*-knockout bacmid, bAc111KO, was constructed via the λ Red recombination system, as described previously [7]. In bAc111KO bacmid, 6 nt of the 5′-end and 118 nt of the 3′-end of *ac111* ORF were retained to avoid affecting the transcription of *ac110* and *ac112*; an 80-bp region of *ac111* was deleted and replaced with a 1038-bp *Cm* cassette (Figure 2A).

The absence of *ac111* and the replacement with the *Cm* gene in AcMNPV bacmid were confirmed by PCR analysis. As shown in Figure 2B, the primers ac111PF4/ac111PR2 produced no PCR product in bAc111KO, while a 420-bp fragment was amplified in AcMNPV bacmid bMON14272. Similarly, the primers ac111PF1/ac111PR4 produced no PCR product in bAc111KO but produced a 480-bp fragment in AcMNPV bacmid bMON14272. The primers CmF/CmR produced no PCR product in AcMNPV bacmid bMON14272, but a 1038-bp fragment was amplified in bAc111KO. Finally, the primers ac111PF1/ac111PR2 produced a 1778-bp fragment in bAc111KO but an 820-bp fragment in AcMNPV bacmid bMON14272. These results demonstrated that the target deletion region of *ac111* was successfully replaced by the *Cm* gene.

In order to facilitate the observation of the viral infection progress, the *polh* gene of AcMNPV and the *gfp* gene were inserted into the *polh* locus of bAc111KO via Tn7-mediated transposition to construct the *ac111*-knockout AcMNPV, vAc111KO, as described previously [45]. To confirm the phenotype observed in vAc111KO resulted from the deletion of *ac111*, a rescue virus, vAc111:HA, was constructed by inserting the *ac111* gene under the control of its native promoter with an HA tag prior to the *ac111* stop codon, along with the *polh* and *gfp* genes, into the *polh* locus of bAc111KO (Figure 2A). Similarly, a wild-type AcMNPV control, vAcWT, was generated by inserting the *polh* and *gfp* genes into bMON14272 (Figure 2A).

### 3.3. Ac111 is Not Required forBV Production

**Figure 3:**
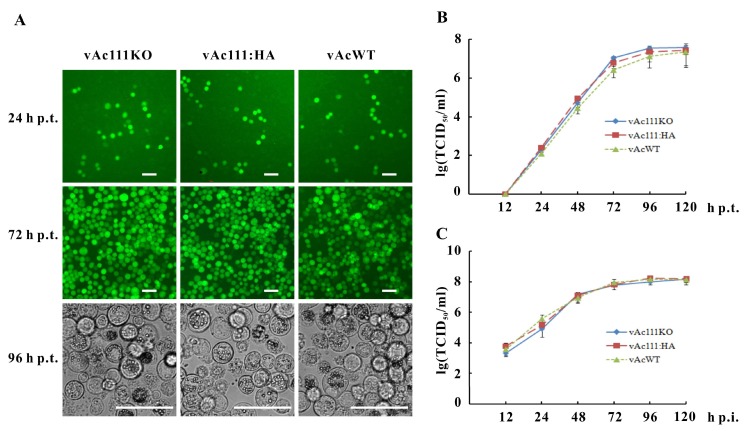
Analysis of viral replication. (**A**) Microscopic analyses of Sf9 cells transfected with vAc111KO, vAc111:HA, or vAcWT bacmid DNA. Fluorescence microscopy revealed the progress of the viral infection for the three viruses at 24 and 72 h p.t. Light microscopy revealed the formation of occlusion bodies (OBs) for the three viruses at 96 h p.t.; scale bar, 50 um; (**B**) Viral growth curves. Sf9 cells were transfected with vAc111KO, vAc111:HA, or vAcWT bacmid DNA (upper graph), or infected with the three viruses (MOI = 5 TCID_50_/cell) (lower graph). The supernatants were harvested at the designated time points and the viral titers were determined using a TCID_50_ endpoint dilution assay. Each data point represents the average of three independent assays; the error bars represent the standard deviations.

To study the effect of *ac111* deletion on viral replication, Sf9 cells were transfected with vAc111KO, vAc111:HA, or vAcWT bacmid DNA, and virus infections were monitored using a fluorescence microscope. As shown in Figure 3A, similar amounts of GFP-positive cells were observed in the three groups at 24 h p.t., indicating comparable transfection efficiencies. By 72 h p.t., almost all vAc111KO-, vAc111:HA-, and vAcWT-transfected cells showed GFP fluorescence, indicating the infection had spread from the cells initially transfected. Light microscopy showed that OBs were produced in the three groups, and there were no obvious differences in the OB production (Figure 3A), suggesting similar infection progress among all three viruses.

To further evaluate the effect of the *ac111* deletion on virus propagation, viral growth curve analysis was performed. Sf9 cells transfected with vAc111KO, vAc111:HA, or vAcWT or infected with BVs showed a normal and steady increase in virus production and similar virus growth kinetics (Figure 3B). In Se301 cells infected with vAc111KO, vAc111:HA, or vAcWT, the three virus growth curves were similar [50]. In addition, no obvious differences were observed in plaque sizes in Sf9 cells transfected with vAc111KO, vAc111:HA, or vAcWT [50].

Taken together, these results demonstrated that the deletion of *ac111* had no apparent effect on viral replication in Sf9 cells or Se301 cells.

### 3.4. Ac111 is Not Required for Viral DNA Replication

**Figure 4:**
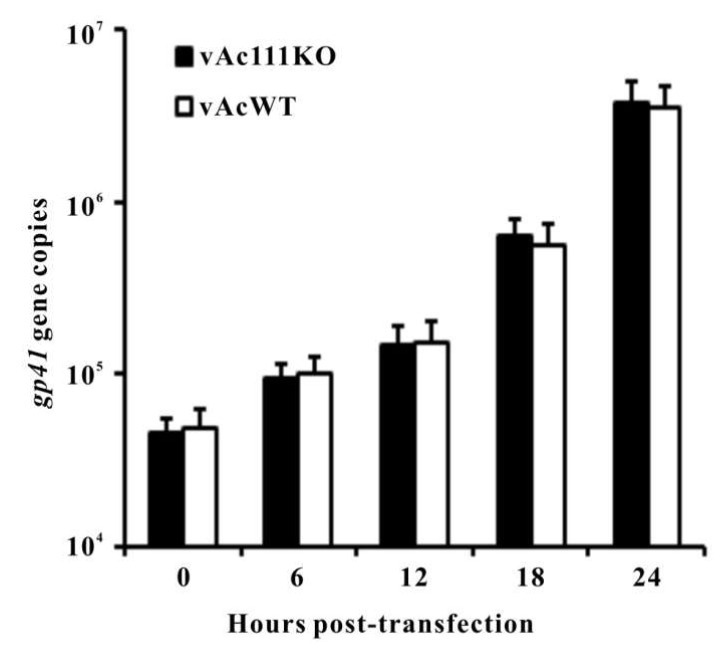
qPCR analysis of viral DNA synthesis in Sf9 cells. Sf9 cells were transfected with vAc111KO or vAcWT bacmid DNA. At the designated time points, total intracellular DNA was extracted, further digested with the restriction enzyme *Dpn*I to eliminate input bacmid DNA, and quantified by qPCR. The values represent the average of three independent replication assays. The error bars indicate standard deviations.

To assess whether *ac111* deletion leads to a defect in viral DNA replication, qPCR was performed to measure the levels of viral DNA synthesis in vAc111KO-transfected cells during a single replication cycle. vAcWT was used as a positive control. Total cellular DNA was isolated from vAc111KO- or vAcWT-transfected cells at the designated time points, and treated with *Dpn*I to eliminate all input bacmid DNA. As shown in Figure 4, viral DNA replication levels of vAc111KO-transfected cells and vAcWT-transfected cells were comparable throughout a 24-h time course. The qPCR results demonstrated that the deletion of *ac111* did not affect the onset or levels of viral DNA synthesis.

### 3.5. Ac111 is Not Essential for Virus Morphogenesis

**Figure 5:**
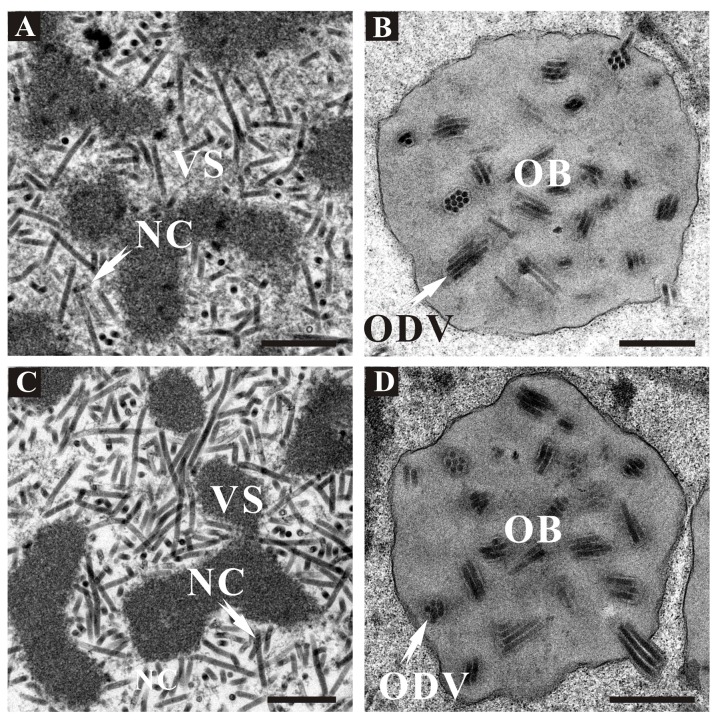
Electron micrographs of Sf9 cells transfected with vAc111:HA (**A**,**B**) or vAc111KO (**C**,**D**) at 72 h p.t. (**A**,**C**) Partial view of the virogenic stroma (VS) at a higher magnification. Normal nucleocapsids (NC) are found in the VS; (**B**,**D**) Image of OB showing embedded multiple-nucleocapsid ODVs. Scale bar, 500 nm.

Electron microscopy observations were performed using thin sections of vAc111KO-, vAcWT-, and vAc111:HA-transfected Sf9 cells at 72 h p.t., to further investigate whether the *ac111* deletion had any effect on virus morphogenesis (Figure 5). In vAc111:HA-transfected Sf9 cells, the typical characteristics were similar to those of vAcWT-transfected cells, including a well-defined virogenic stroma (VS) enriched with electron-dense, rod-shaped nucleocapsids in the center of the nucleus (Figure 5A), and developing OBs embedding numerous mature ODVs in the ring zone (Figure 5B). The cells transfected with vAc111KO exhibited morphologically indistinguishable characteristics to those of vAc111:HA and vAcWT (Figure 5C,D). These results indicated that the deletion of *ac111* did not affect virus morphogenesis.

### 3.6. Ac111 Plays a Role in Oral Infectivity in a Host-Dependent Manner

To determine the effect of *ac111* deletion on the infectivity of AcMNPV in insect larvae, bioassays with BVs and OBs grown in Sf9 cells were performed in two host species, *T. ni* and *S. exigua*. Intrahemocoelic injection of vAc111KO BV supernatant into fourth-instar *T. ni* at 1 TCID_50_ units/larva produced 67% mortality, which was not significantly different from that of vAc111:HA (63%) or vAcWT (63%) (Student’s *t*-test, *p*> 0.05) (Table 2). Intrahemocoelic injection of vAc111KO, vAc111:HA or vAcWT BV supernatant into fourth-instar *S. exigua* at 2 TCID_50_ units/larva produced 70%, 67%, or 70% mortalities, respectively, and there was no significant difference among the mortalities (Student’s *t*-test, *p*> 0.05) (Table 2). However, bioassays with OBs revealed that *ac111* affected the oral infectivity of AcMNPV in the two host species in a different way. Bioassays with OBs in *T. ni* larvae showed that, the LD_50_ values of vAc111:HA and vAcWT were 59 and 54 OBs/larva, respectively with overlapping 95% confidence interval indicating they were not significantly different, the LD_50_ of vAc111KO (300 OBs/larva) was approximately five times more than that of vAcWT (Table 2). The LT_50_ value of vAc111:HA (86 h) was comparable to that of vAcWT (85 h), but the LT_50_ value of vAc111KO (106 h) was 21 h longer than that of vAcWT (Table 2). Therefore, the deletion of *ac111* reduced the infectivity and killing speed of AcMNPV in *T. ni* larvae. However, in *S. exigua* larvae, no significant differences in infectivity or killing speed were observed among vAc111KO, vAc111:HA and vAcWT, with the LD_50_ values being 5006, 4650, and 4535 OBs/larva, and the LT_50_ values being 148 h, 141 h and 140 h, respectively (as determined by overlapping 95% confidence interval) (Table 2). This suggests that the deletion of *ac111* did not impact *per os* infectivity of AcMNPV in *S. exigua* larvae.

Taken together, the bioassays demonstrated that *ac111* plays a role in primary oral infection of AcMNPV and the effect is host dependent.

## 4. Discussion

*ac111* is one of the highly conserved lepidopteran-specific baculovirus genes, but its role in the AcMNPV replication remains unknown. In this study, we demonstrated that *ac111* is important for AcMNPV to efficiently establish *per os* infection in *T. ni* larvae specifically, while is dispensable for *per os* infectivity of AcMNPV in *S. exigua* larvae, thus providing evidence for its important role as a conserved baculovirus gene.

To investigate the function of *ac111* in the AcMNPV life cycle, an *ac111*-knockout virus was generated and characterized. We found that *ac111* was dispensable for virus replication in vitro as evidenced by indistinguishable characteristics such as a normal viral growth curve, normal plaque, steady viral DNA replication and normal virus morphogenesis, compared to those of vAcWT. These results were consistent with the study on *Bm93* [33]. Bioassays showed that there was no significant difference between the LD_50_ and LT_50_ of vAc111KO OBs and those of vAcWT in *S. exigua* larvae. However, the LD_50_ of vAc111KO OBs was approximate five times higher than that of vAcWT OBs in *T. ni* larvae, and the LT_50_ of vAc111KO was 21 h longer than that of vAcWT in *T. ni* larvae. Furthermore, the deletion of *ac111* did not affect BV infectivity through intrahemocoelic injection in *T. ni* and *S. exigua* larvae. Taken together, these results indicated that *ac111* affects the *per os* infectivity of AcMNPV in a host-dependent manner.

Of the 154 putative ORFs encoded in the AcMNPV genome, the function of more than 70% of these ORFs has been determined. While some genes were found to be nonessential and could be deleted without affecting virus replication, some genes are essential for virus replication in vitro and participate in critical steps in the virus life cycle, such as DNA replication, early/late gene transcription, or nucleocapsid assembly. There are some other genes that are dispensable for virus replication in vitro but function during oral infection of insect larvae. Genes belonging to this category include the nine *pifs* and several virus virulence-related genes, such as *ac111*, *ac145*, and *ac150*. Baculoviruses initiate infection orally when their insect hosts consume and digest OBs in the alkaline pH of the midgut, releasing ODVs to infect the midgut epithelial cells. A number of viral membrane proteins, including the PIF proteins, have been identified to be essential for this process. Considering the pivotal role of PIFs, these proteins are encoded by baculovirus core genes [6], indicating that they play pivotal roles in *per os* infection of all baculoviruses and thus have been conserved during baculovirus evolution. Other virus virulence-related genes are only conserved in certain baculoviruses, implying that they might function distinctly compared to PIFs in a host dependent manner and facilitate the efficient establishment of baculovirus infection in specific hosts.

Similar to *ac111*, previous studies have demonstrated that two AcMNPV genes, *ac145* and *ac150* affect oral infectivity in a host dependent manner [26]. *ac145* and *ac150* encode proteins related to the 11K proteins with a characteristic C_6_ motif of conserved cysteine residues in a well-defined spacing pattern. Previous studies have found that the deletion of *ac145* led to a six-fold drop in infectivity of AcMNPV in *T. ni* larvae but not in *Heliothis virescens* larvae [26]. On the other hand, the deletion of *ac150* had no effect on infectivity of AcMNPV in either host [26]. However, the deletion of both *ac145* and *ac150* significantly reduced the infectivity of AcMNPV (39-fold) in *H. virescens* larvae [26]. In another study, significantly more OBs were required per LD_50_ for *ac150* deletion AcMNPVcompared with wild-type virus in *H. virescens*, *S. exigua*, and *T. ni* larvae (4.1-, 5.6-, and 18-fold increases, respectively), suggesting that Ac150 indeed had an enhancing effect on *per os* infection and that the effect was host-specific [31]. In this study, we showed that *ac111* specifically affected viral infectivity and killing speed of AcMNPV in *T. ni* larvae but is dispensable in *S. exigua* larvae. Therefore, although AcMNPV could establish infection in *T. ni*, *S. exigua*, and *H. virescens* larvae, differences might exist that subtly affect the fitness of AcMNPV in these different insect hosts. It is intriguing to speculate that a group of proteins in AcMNPV, such as Ac111, Ac145, and Ac150, might specifically affect the infectivity of a virus in different hosts. We also cannot rule out the possibility that these viral proteins might interact with each other or with specific host proteins, and these interactions may eventually determine the host tropism of AcMNPV. Further studies are needed to verify this assumption.

Taken together, this study has shown that *ac111* was not required for AcMNPV replication in vitro, but it played a pivotal role in the viral infectivity in vivo in *T. ni* but not in *S. exigua* larvae. Further experiments are needed to unravel the specific role of *ac111* during Ac*M*NPV infection in *T. ni* larvae. These studies will likely shed light on our understanding of the mechanism of baculovirus–insect interactions, to ascertain the fitness of baculoviruses in different hosts.

## Figures and Tables

**Table 1:** Primer sequences used in this study.

Applications	Primer	Sequence
Amplification of *ac111* 5′ flanking sequences	ac111PF1	5′-GAGCTCGTTTCCATAATAATGAGGATACGCT-3′
	ac111PR1	5′-GGATCCATCCATAGTGCAGCACAGGCAAT-3′
Amplification of *ac111* 3′ flanking sequences	ac111PF2	5′-CTGCAGATCGGTGTACGAAGATGTCACATATA-3′
	ac111PR2	5′-AAGCTTAGATTATTTTAATTTGTGAACTCGTACC-3′
Amplification of the 5′ end of the *ac111* native promoter and ORF	ac111PF3	5′-GAGCTCTACAAAACGATGCATTTATAGCGC-3′
Amplification of the 3′ end of *ac111* tagged with an HA	ac111PR3	5′-GGATCCTTAGGCGTAATCTGGGACGTCGTATGGGTA- TTTATATTTGTTTTCTTTGTTATAACCG-3′
Amplification of the 5′ end of *ac111* target deletion region	ac111PF4	5′-AATTATTCGGTGCAAAATTTTTACAAC-3′
Amplification of the 3′ end of *ac111* target deletion region	ac111PR4	5′-TTCTTGATGTTACCATCGTGAAGCGTTG-3′
Amplification of *Cm* cassette	CmPF	5′-CATGTCTGGATCCTTCGAATAAATACCTGTGACGG-3′
	CmPR	5′-GGATTCTAAACCAGCAATAGACATAAGCGGC-3’
Real-time PCR, specific for AcMNPV DNA	gp41-F	5′-CGTAGTGGTAGTAATCGCCGC-3′
	gp41-R	5′-AGTCGAGTCGCGTCGCTTT-3′

**Table 2:** Dose-mortality and time-mortality responses of insect larvae infected with different viruses.

Inoculation Methods and Viruses or Control	Insect Larvae *^a^*	Dose	Mortality at 7 Days p.i. (%)	Dose-Mortality Regression			LT_50_ (hr) (95% Confidence Interval)
				LD_50_*^c^* (95% Confidence Interval)	slope ± SE	Chi^2^*^d^*/df*^e^*	
Injection							
vAcWT	*T. ni*	1 TCID_50_	63				
vAc111:HA	*T. ni*	1 TCID_50_	63				
vAc111KO	*T. ni*	1 TCID_50_	67				
vAcWT	*S. exigua*	2 TCID_50_	70				
vAc111:HA	*S. exigua*	2 TCID_50_	67				
vAc111KO	*S. exigua*	2 TCID_50_	70				
H_2_O	*T. ni* *S. exigua*		3.3*^b^*				
*Per os*							
vAcWT	*T. ni*			54 (34–84)	1.33 ± 0.22	4.80/3	85 (81–90)
vAc111:HA	*T. ni*			59 (36–92)	1.37 ± 0.22	4.76/3	86 (82–91)
vAc111KO	*T. ni*			300 (187–482)	1.39 ± 0.28	1.82/2	106 (101–112)
vAcWT	*S. exigua*			4535 (2823–7283)	1.13 ± 0.18	7.72/3	140 (126–155)
vAc111:HA	*S. exigua*			4650 (2989–6877)	1.42 ± 0.20	2.59/3	141 (129–154)
vAc111KO	*S. exigua*			5006 (2975–8423)	1.51 ± 0.21	2.21/3	148 (133–164)

*^a^* A total of 30 larvae were used for each treatment. *^b^* Larval death not due to virus infection (microscopy and PCR analysis). *^c^* Expressed as OBs/larva. *^d^* Heterogeneity of deviations from model. *^e^* Number of degrees of freedom of Chi^2^ values.
